# Supplementary material for: Controls on the southwest USA hydroclimate over the last six glacial-interglacial cycles
Source: Nat Commun. 2025 Nov 14;16:10007. doi: 10.1038/s41467-025-64963-1 (PMC12618935; doi:10.1038/s41467-025-64963-1)
Supplement: Supplementary file 1 — Supplementary Information [file 41467_2025_64963_MOESM1_ESM.pdf]

Supplementary materials and figures for manuscript "Controls on the southwest USA hydroclimate over the last six glacial-interglacial cycles**"**

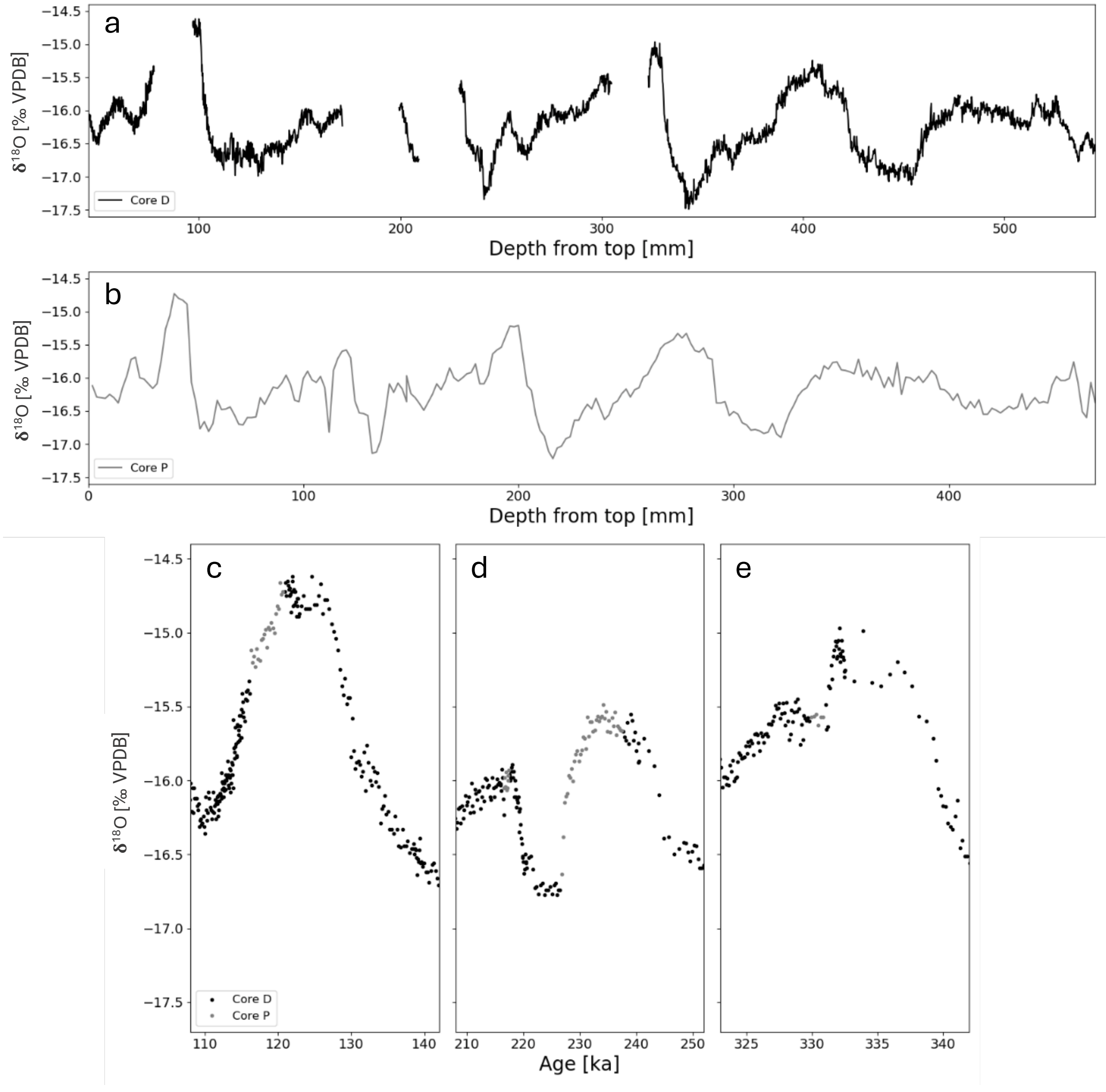

Figure S1: Splicing DH2 $\delta^{18}O$ records. Addition of data from a deeper core (Core P) to fill the gaps in the $\delta^{18}O$ record (Core D). A: $\delta^{18}O$ against depth from top with four gaps due a low water table. B: Low resolution $\delta^{18}O$ record of a deeper core (Core P). This low-resolution record was used to identify the correct time intervals. C-E: The core D record (black) is patched with data from core P (grey). $\delta^{18}O$ of core P were offset by up to 0.25 ‰ to align with the core D record (see methods).

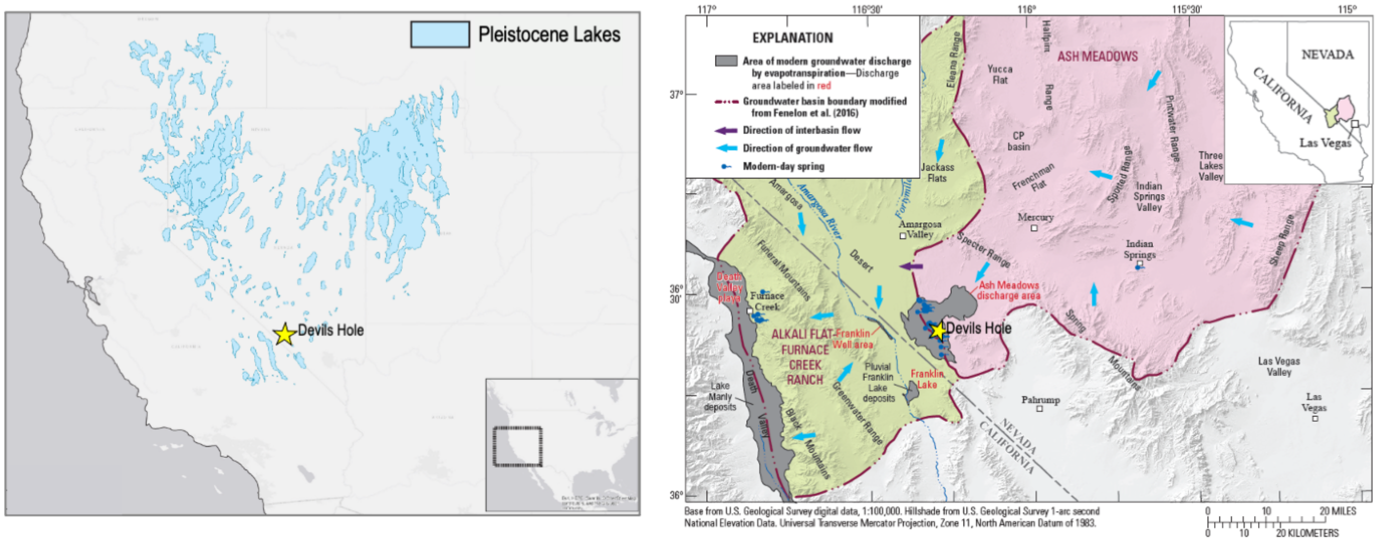

Figure S2: (left) southwest United States Great Basin. Devils Hole and Devils Hole 2 caves (200 m apart) indicated by yellow star. (Right) study site and groundwater flow map adapted from (*1*).

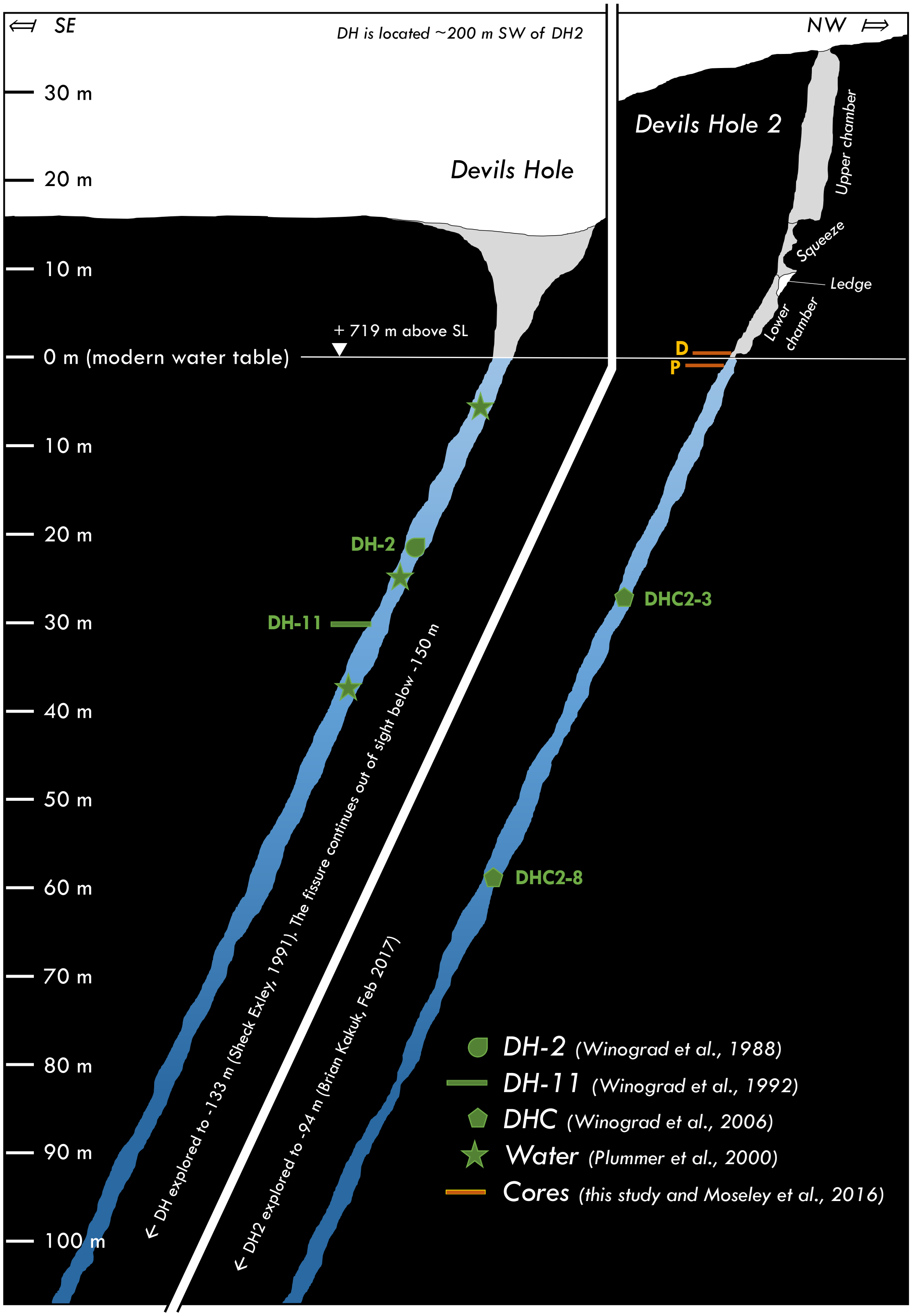

Figure S3: Schematic cross section of Devils Hole (DH) and Devils Hole 2 (DH2) caves. Location of core-D (+1.8 m) and core-P (-1.6 m) is indicated by orange lines. Figure courtesy of Mathieu Pythoud.

Table S1: **U-series results.** ^{230}Th-^{234}U ages from Moseley, Edwards, Wendt, Cheng, Dublyansky, Lu, Boch and Spötl (*2*), Wendt, Pythoud, Moseley, Dublyansky, Edwards and Spötl (*3*), Li, Wendt, Dublyansky, Moseley, Spötl and Edwards (*4*) and this study (italicized). Analytical errors are 2σ of the mean. DFT= distance from top of core. U decay constants: λ_{238} = 1.55125×10^{-10} and λ_{234} = 2.82206×10^{-6} (*5, 6*). Th decay constant: λ_{230} = 9.1705×10^{-6} (*5*). Age corrections were calculated using an average crustal ^{230}Th/^{232}Th atomic ratio of 4.4×10^{-6} ± 2.2×10^{-6}. Selected values represent material at secular equilibrium, with the crustal ^{232}Th/^{238}U value of 3.8. *δ^{234}U = ([^{234}U/^{238}U]$_{activity}$-1) ×1000. **δ^{234}U$_{inital}$ are back-calculated to initial values based on respective U-Th corrected age. ***B.P. stands for "Before Present" where the "Present" is defined as the year 1950 CE.[I] = Replicate.

DFT (cm)	^{238}U (ppb)	^{232}Th (ppt)	^{230}Th / ^{232}Th (atomic x10^{-6})	δ^{234}U* (measured)	^{230}Th / ^{238}U (activity)	^{230}Th Age (yr) (uncorrected)	δ^{234}U$_{Initial}$** (corrected)	^{230}Th Age (yr) (corrected)	^{230}Th Age (yr BP)*** (corrected BP)
0.00	1906.5±2.0	10850.3± 217.5	356±7	1,716.9± 2.4	0.12282±0.00035	5,021±15	1741.1±2.4	4961	**4891±45**
0.09	1718.3±3.6	4573.9± 100.5	1203±28	1,713.2± 2.4	0.19419±0.00168	8,038±72	1752.4±2.5	8010	**7940±75**
0.23	1502.4±1.6	16146.9± 323.6	303±6	1,707.5± 2.8	0.19767±0.00050	8,205±23	1746.9±2.8	8091	**8021±84**
0.26	1417.7±1.5	18682.6± 374.5	247±5	1,705.1± 2.3	0.19777±0.00069	8,217±30	1744.4±2.4	8077	**8007±103**
0.32	1427.8±1.4	6092.8± 122.2	828±17	1,706.3± 2.1	0.21420±0.00044	8,918±20	1749.6±2.1	8873	**8803±38**
0.38	1047.3±1.2	12682.5± 254.1	422±8	1,705.4± 2.5	0.31026±0.00066	13,121±32	1769.1±2.6	12994	**12924±95**
0.40	492.4±0.6	8789.9± 176.1	556±11	1,675.7± 2.9	0.60221±0.00118	27,078±67	1807.8±3.2	26893	**26823±147**
0.58	467.4±0.4	5372.8± 109.0	881±18	1,714.5± 2.4	0.61401±0.00230	27,224±116	1850.7±2.7	27106	**27036±143**
0.70	482.1±0.4	5888.3± 118.1	881±18	1,667.6± 2.2	0.65234±0.00135	29,699±74	1812.7±2.4	29572	**29502±116**
1.02	494.6±0.5	6944.2± 139.3	876±18	1,641.7± 2.5	0.74556±0.00204	34,920±115	1811.0±2.8	34775	**34705±154**
1.48	505.5±0.5	10089.4± 202.2	736±15	1,612.1± 2.6	0.89113±0.00196	43,514±124	1821.7±3.1	43308	**43238±191**
1.89	469.5±0.4	3970.0± 79.6	1935±39	1,572.9± 2.2	0.99251±0.00170	50,415±118	1813.0±2.7	50327	**50257±133**
2.24	469.4±0.4	4535.4± 91.0	1793±36	1,540.8± 2.3	1.05096±0.00192	54,926±139	1798.7±2.8	54826	**54756±156**
2.59	534.1±0.5	4019.2± 80.7	2445±49	1,513.9± 2.4	1.11566±0.00205	59,985±158	1792.7±3.0	59907	**59837±167**
2.99	524.3±0.5	2596.4± 52.2	3852±78	1,502.8± 2.2	1.15707±0.00194	63,184±153	1795.8±2.7	63132	**63062±158**
3.39	447.4±0.5	1698.9± 34.3	5308±107	1,483.9± 2.3	1.22236±0.00209	68,502±176	1800.1±3.0	68462	**68392±178**
3.72	480.6±0.5	3022.2± 60.7	3333±67	1,454.0± 2.4	1.27135±0.00220	73,334±195	1788.0±3.1	73268	**73198±200**
4.08	476.1±0.4	3125.5± 62.7	3287±66	1,428.8± 2.3	1.30848±0.00214	77,317±199	1776.8±3.0	77249	**77179±204**
4.46	467.2±0.5	7245.1± 145.3	1448±29	1,395.5± 2.3	1.36222±0.00253	83,314±243	1764.5±3.2	83150	**83080±269**
4.98	419.2±0.6	2074.6± 42.0	4665±95	1,365.7± 2.7	1.40029±0.00346	88,187±343	1751.3±3.9	88135	**88065±344**
5.39	405.4±0.5	482.0± 11.1	19976±461	1,321.1± 2.4	1.44045±0.00324	94,489±342	1724.7±3.5	94476	**94406±342**
5.74	384.3±0.4	423.2± 10.8	22230±569	1,298.0± 2.1	1.48463±0.00351	100,314±381	1722.7±3.4	100302	**100232±381**
5.99	427.9±0.5	272.2± 5.7	38502±814	1,292.7± 2.5	1.48572±0.00253	100,783±308	1718.0±3.6	100776	**100706±308**
6.14	409.3±0.4	247.5± 6.9	41028±1142	1,272.5± 1.9	1.50465±0.00260	104,171±307	1707.4±3.0	104164	**104094±307**
6.54	425.8±0.4	9407.0± 188.5	1139±23	1,257.9± 2.1	1.52651±0.00276	107,590±338	1703.1±3.3	107352	**107282±377**
6.94	415.8±0.4	244.0± 6.9	43463±1223	1,236.7± 2.0	1.54700±0.00280	111,552±356	1694.2±3.2	111545	**111475±356**
7.77	602.4±0.7	122.7± 7.0	126477±7194	1,204.8± 2.2	1.56305±0.00309	116,139±415	1672.0±3.6	116137	**116067±415**
9.74	1054.4±1.3	91.5± 2.6	297873±8494	1,153.2± 2.3	1.56712±0.00273	121,465±411	1624.7±3.7	121464	**121394±411**
9.94	511.3±0.4	72.2± 2.5	185162±6478	1,163.2± 1.7	1.58641±0.00196	122,928±304	1645.5±2.8	122926	**122856±304**
10.14	564.0±0.6	533.3± 11.4	27891±595	1,123.2± 2.1	1.59950±0.00258	128,826±421	1615.6±3.6	128816	**128746±421**
10.29	540.4±0.5	2461.1± 49.5	5869±118	1,163.1± 2.0	1.62100±0.00229	127,406±368	1666.2±3.4	127356	**127286±369**
10.44	530.8±0.5	1816.2± 36.5	8147±164	1,180.7± 2.2	1.69073±0.00244	134,858±418	1727.4±3.8	134821	**134751±418**
10.59	525.9±0.5	795.7± 17.0	18618±398	1,170.6± 2.0	1.70859±0.00288	138,550±476	1730.6±3.8	138533	**138463±476**
10.74	522.0±0.5	745.2± 15.2	19806±406	1,167.5± 2.1	1.71484±0.00253	139,819±442	1732.2±3.8	139804	**139734±443**
10.89	500.6±0.5	365.7± 7.8	39099±834	1,161.6± 2.0	1.73223±0.00250	143,095±451	1739.4±3.7	143087	**143017±451**
10.99	518.3±0.5	606.8± 13.2	24486±534	1,150.4± 2.0	1.73863±0.00316	145,493±547	1734.5±4.1	145480	**145410±547**
11.44	546.0±0.5	482.3± 10.6	32990±729	1,130.4± 2.0	1.76711±0.00279	152,714±537	1739.4±4.1	152704	**152634±537**
11.74	524.5±0.5	1275.3± 26.0	12027±245	1,127.8± 2.0	1.77365±0.00259	154,151±517	1742.4±4.0	154125	**154055±517**
11.74[I]	545.7±0.5	1309.8± 27.2	12200±253	1,141.0± 2.2	1.77595±0.00280	152,607±550	1755.1±4.3	152581	**152511±550**
11.94	510.5±0.5	1188.6± 24.4	12727±262	1,128.9± 2.1	1.79727±0.00355	157,902±675	1762.6±4.6	157877	**157807±675**
12.44	534.2±0.5	3333.8± 67.0	4790±96	1,115.4± 1.9	1.81289±0.00295	162,731±605	1765.1±4.3	162665	**162595±607**
12.84	485.3±0.5	1322.6± 27.1	11050±227	1,096.3± 1.9	1.82647±0.00332	168,407±703	1763.1±4.7	168378	**168308±703**
13.24	585.1±0.6	5629.1± 112.8	3143±63	1,078.6± 2.0	1.83389±0.00293	173,000±675	1757.0±4.0	172898	**172828±679**
13.78	555.6±0.5	2890.1± 58.0	5950±120	1,091.3± 1.9	1.87707±0.00282	179,063±675	1808.6±4.6	179009	**178939±676**
14.18	442.2±0.4	6711.1± 134.5	2064±41	1,084.0± 1.9	1.90006±0.00297	185,284±738	1827.8±5.0	185127	**185057±745**

14.58	362.8±0.3	1676.2± 34.0	6788±138	1,062.9± 2.0	1.90203±0.00325	190,300±852	1818.3±5.6	190252	**190182±853**
15.04	378.9±0.3	1334.6± 27.1	8921±182	1,050.4± 2.1	1.90569±0.00330	193,985±904	1815.8±5.9	193949	**193879±904**
15.30	363.0±0.4	1650.6± 33.2	6932±140	1,050.0± 2.0	1.91180±0.00296	195,479±834	1822.8±5.5	195432	**195362±834**
15.30[I]	383.1±0.3	1946.2± 40.1	6216±128	1,045.9± 2.1	1.91545±0.00347	197,324±967	1825.1±6.2	197271	**197201±968**
15.54	407.6±0.4	1837.0± 36.9	6946±140	1,024.8± 2.2	1.8987±0.00313	198,544±936	1794.3±6.1	198497	**198427±937**
15.84	430.3±0.3	1879.3± 38.0	7208±146	1,012.1± 1.9	1.9092±0.00303	204,412±918	1801.7±5.8	204366	**204296±918**
16.98	*852.3±1.6*	*3347.3± 67.6*	*8026±164*	*971.1± 2.6*	*1.9118±0.00626*	*216,842±1957*	*1790.5±11.0*	*216801*	***216731±1957***
20.02	424.7±0.5	1979.5± 40.1	6722±137	957.2±1.9	1.9002±0.0040	217856±1307	1769.8±7.4	217806	**217736±1307**
20.56	521.1±0.7	7271.6± 146.2	2232±45	939.2±2.1	1.8889±0.0051	220263±1678	1747.9±9.1	220115	**220045±1680**
20.86	407.6±0.5	3179.9± 64.1	3992±81	921.7±2.2	1.8891±0.0044	226235±1571	1744.8±8.7	226151	**226081±1571**
23.00	506.4±0.6	2366.5± 47.9	6666±136	889.0±2.0	1.8893±0.0043	238447±1711	1742.2±9.3	238397	**238327±1711**
23.12	484.0±0.6	2942.0± 59.4	5115±104	877.1±1.9	1.8859±0.0041	242064±1704	1736.4±9.2	241999	**241929±1704**
23.16	498.7±0.6	4418.8± 88.7	3498±70	878.3± 2.0	1.8797±0.00363	239,347±1541	1725.2±8.5	239252	**239182±1541**
23.34	551.0±0.6	9039.1± 181.0	1907±38	872.3± 2.1	1.8972±0.00294	248,412±1476	1757.5±8.5	248238	**248168±1479**
23.34[I]	573.9±0.7	8824.0± 177.1	2038±41	873.2± 2.0	1.9008±0.00412	249,399±1829	1764.3±10.0	249236	**249166±1830**
23.70	484.3±0.6	2791.8± 56.3	5445±110	863.9± 2.0	1.9037±0.00418	254,806±1957	1772.8±10.6	254745	**254675±1956**
23.96	512.1±0.6	6432.4± 129.1	2512±51	842.4± 1.9	1.9139±0.00393	270,149±2109	1804.9±11.5	270018	**269948±2109**
24.62	487.0±0.6	1758.4± 35.9	8789±180	837.6± 2.1	1.9246±0.00422	277,989±2409	1835.3±13.3	277951	**277881±2409**
24.84	418.1±0.5	3099.3± 62.6	4242±86	826.7± 2.1	1.9075±0.00464	275,960±2567	1800.8±13.8	275882	**275812±2566**
25.42	443.3±0.5	2300.4± 46.4	6042±122	800.9± 1.9	1.9017±0.00386	289,058±2482	1810.4±13.4	289003	**288933±2481**
26.38	436.3±0.5	5356.6± 107.5	2527±51	768.7± 2.0	1.8814±0.00346	300,111±2605	1792.3±14.0	299981	**299911±2603**
27.18	390.8±0.4	1104.5± 22.9	10870±226	742.5± 1.8	1.8631±0.00342	309,070±2748	1776.1±14.5	309039	**308969±2747**
28.18	399.8±0.4	13709.4± 274.6	888±18	717.2± 1.8	1.8470±0.00332	320,007±2984	1767.7±15.6	319645	**319575±2987**
28.74	408.3±0.6	7518.4± 360.7	1645±79	707.7± 2.9	1.8370±0.00596	321,469±5290	1752.3±27.1	321272	**321202±5284**
29.18	458.0±0.5	3236.7± 65.1	4257±86	695.3± 1.8	1.8245±0.00349	323,866±3218	1733.8±16.4	323790	**323720±3217**
30.00	153.9±0.1	276.2± 8.4	16777±512	692.4± 1.8	1.8259±0.00413	327,802±3779	1746.1±19.2	327781	**327711±3778**
32.50	1083.5±1.7	580.4± 12.5	55708±1203	668.7± 1.9	1.8100±0.00439	339,887±4494	1745.0±22.7	339880	**339810±4494**
32.84	570.7±0.6	658.4± 13.3	25613±517	666.1± 1.7	1.7921±0.00238	327,348±2606	1677.8±13.0	327334	**327264±2606**
32.94	569.9±0.7	1753.8± 35.2	9617±193	660.0± 2.0	1.7949±0.00319	336,407±3596	1705.2±18.1	336373	**336303±3596**
33.06	561.0±0.7	2359.8± 47.5	7057±142	659.3±2.0	1.8004±0.0040	342,057±4321	1730.9±21.7	342011	**341941±4319**
33.36	668.0±0.9	2999.2± 60.4	6697±136	677.2±1.8	1.8237±0.0049	342,437±4866	1779.7±24.9	342388	**342318±4864**
33.54	643.9±1.0	2649.0± 53.7	7284±149	672.3±2.2	1.8177±0.0065	342,672±6480	1768.0±32.8	342627	**342557±6478**
33.72	586.9±0.8	3285.6± 66.0	5376±109	675.5±1.9	1.8254±0.0052	345,951±5366	1792.9±27.6	345891	**345821±5364**
34.40	573.2±0.8	1121.7± 22.7	15300±310	663.6±1.9	1.8158±0.0042	351,472±4778	1789.1±24.6	351450	**351380±4777**
34.72	642.1±0.7	997.1± 20.0	19263±388	659.8±1.8	1.8143±0.0025	354,762±3406	1795.6±17.9	354745	**354675±3405**
35.10	526.4±0.5	641.3± 12.9	24552±496	651.9±1.7	1.8140±0.0026	365,414±3752	1828.1±19.9	365400	**365330±3751**
35.40	504.3±0.8	398.2± 8.9	37892±853	648.8±2.2	1.8144±0.0050	370,428±6623	1845.3±35.0	370418	**370348±6623**
35.50	502.2±0.7	248.1± 5.8	60475±1411	644.7±1.7	1.8121±0.0045	373,772±5975	1851.3±31.6	373765	**373695±5975**
36.58	453.4±0.6	292.1± 6.6	45708±1037	620.2±1.9	1.7860±0.0039	381,296±5899	1819.0±30.8	381288	**381218±5899**
39.04	505.9±0.6	20.2± 2.7	715239±94502	578.7±1.8	1.7359±0.0039	387,590±6349	1727.7±31.4	387517	**387447±6348**
40.08	488.9±0.6	3888.2± 78.0	3546±71	552.3±1.8	1.7104±0.0035	401,751±6811	1715.6±33.4	401664	**401594±6807**
40.54	514.3±2.8	810.9± 4.5	17950±14	554.3±0.8	1.7164±0.0016	407,217±3101	1749.0±15.5	407207	**407137±3101**
41.70	481.3±0.6	631.0± 12.9	21305±436	531.5±1.7	1.6940±0.0035	421,530±7878	1746.4±39.2	421513	**421443±7877**
42.08	537.9±0.7	730.7± 15.0	20415±420	519.9±1.6	1.6819±0.0037	428,657±8693	1742.8±43.1	428640	**428570±8692**
42.24	614.3±0.9	492.0± 10.3	34482±725	514.1±1.9	1.6752±0.0036	430,706±9227	1733.5±45.6	430694	**430624±9226**
42.60	651.0±0.7	889.3± 18.0	20293±411	516.6±1.6	1.6812±0.0026	436,257±7414	1769.4±37.5	436239	**436169±7413**
43.90	574.5±0.9	240.4± 5.5	65955±1518	506.8±1.8	1.6736±0.0041	449,250±11458	1800.6±58.7	449242	**449172±11457**
46.52	489.4±2.3	50.7± 0.2	263599±318	487.8±1.0	1.6574±0.0019	475,568±6934	1805.7±48.7	475578	**475508±6934**
47.50	461.0±0.5	84.5± 2.4	147030±4134	471.7± 1.9	1.6355±0.00264	476,009±11360	1807.5±58.5	476002	**475932±11360**
49.40	485.5±0.5	37.7± 2.5	342178±22387	448.9± 1.7	1.6118±0.00212	502,881±12006	1855.4±63.3	502875	**502805±12228**
51.98	447.5±0.1	192.1± 0.1	61314±159	437.8±0.7	1.5965±0.0020	503,197±8473	1811.2±43.4	503206	**503136±8474**
53.48	531.7±0.1	199.4± 0.1	68374±154	402.8±0.4	1.5552±0.0015	536,546±8616	1830.8±44.6	536561	**536491±8617**
54.24	532.9±0.1	177.6± 0.1	76673±207	397.2±0.4	1.5496±0.0019	548,883±11904	1869.4±62.9	548900	**548830±11906**
56.20	389.6±0.4	31.8± 2.1	306762±19876	373.5±1.7	1.5191±0.0021	565,805±21928	1844.0±114.9	559450	**559380±13438**
56.94	320.0±0.3	53.2± 2.0	149855±5625	366.2±1.7	1.5102±0.0024	575,443±25308	1857.6±133.7	561160	**561090±13673**
58.34	542.6±0.6	35.4± 2.7	375952±29151	349.0±1.7	1.4877±0.0027	590,019±30745	1844.5±161.6	575909	**575839±13791**

Table S2: **^{234}U-^{238}U ages** from Li, Wendt, Dublyansky, Moseley, Spötl and Edwards (*4*) and this study (italicized). Analytical errors are 2σ of the mean. DFT= distance from top of core. U decay constants: $\lambda_{238} = 1.55125\times10^{-10}$ and $\lambda_{234} = 2.82206\times10^{-6}$ (*5, 6*). *$\delta^{234}U = ([^{234}U/^{238}U]_{activity}-1) \times1000$. **$\delta^{234}U_{inital}$ are statistically derived using methods outlined in Li, Wendt, Dublyansky, Moseley, Spötl and Edwards (*4*). ***B.P. stands for "Before Present" where the "Present" is defined as the year 1950 CE.

DFT (cm)	^{238}U (ppb)	^{232}Th (ppt)	^{230}Th / ^{232}Th (atomic x10^{-6})	δ^{234}U* (measured)	^{230}Th / ^{238}U (activity)	$\delta^{234}U_{inital}$ (statistically derived)	**^{234}U Age (yr BP)*** (corrected BP)**	δ^{18}O ‰	δ^{13}C ‰
59.08	*562.2±0.9*	*53.9± 1.8*	*234977±7803*	*266.1±1.5*	*1.3677±0.0032*	*1785.31±60.54*	***674160±14480***	*-16.57*	*-1.95*
59.38	*611.8±1.0*	*18.6± 1.8*	*742372±73665*	*274.9±1.8*	*1.3717±0.0030*	*1787.019±60.54*	***663067±14938***	*-16.79*	*-2.04*
59.58	*590.4±0.8*	*42.2± 2.0*	*316043±15122*	*263.2±1.6*	*1.3687±0.0026*	*1782.01±60.54*	***677445±14821***	*-16.85*	*-2.13*
60.82	76.7±0.1	19.4± 1.7	89710±8042	262.7±1.7	1.378±0.0035	1773.28±60.54	**676425±14439**	-16.22	-1.9
61.18	496.6±0.5	28.9± 2.2	389968±29610	262.1±1.9	1.3772±0.0022	1780.12±60.54	**678564±14661**	-16.19	-1.81
61.82	306.2±0.5	98.8± 3.0	86422±2637	257.7±2.2	1.6921±0.0042	1762.15±60.54	**680980±15201**	-15.96	-1.9
62.34	320.9±0.5	83.6± 2.6	86504±2746	247.6±1.6	1.3662±0.0031	1740.36±60.54	**690688±14610**	-15.66	-2
62.56	335.7±0.5	110.6± 3.6	86550±2808	245.6±2.0	1.7299±0.0040	1719.89±60.54	**689460±15390**	-15.53	-2.17
63	404±0.4	1021.3± 20.5	8746±176	241±1.5	1.3407±0.0021	1738.38±60.54	**699827±14556**	-15.6	-1.99
63.4	366.2±0.5	109.9± 3.0	86174±2331	232.7±1.7	1.5685±0.0034	1773.11±60.54	**719313±14700**	-16.29	-1.95
63.8	480.6±0.6	311.3± 6.4	33860±697	229.4±1.5	1.3301±0.0022	1799.00±60.54	**729579±14222**	-16.74	-1.87
63.98	353.5±0.6	94.9± 3.7	86950±3381	228.9±1.9	1.416±0.0034	1780.03±60.54	**726510±14922**	-16.13	-1.78
65.2	353.4±0.5	110.3± 4.1	86852±3236	229.4±1.8	1.6443±0.0038	1767.80±60.54	**723277±14859**	-16.12	-1.92

Table S3: **Eliminated ^{230}Th-^{234}U results (see text).** Analytical errors are 2σ of the mean. DFT= distance from top of core. U decay constants: $\lambda_{238} = 1.55125\times10^{-10}$ and $\lambda_{234} = 2.82206\times10^{-6}$ (*5, 6*). Th decay constant: $\lambda_{230} = 9.1705\times10^{-6}$ (*5*). Age corrections were calculated using an average crustal $^{230}Th/^{232}Th$ atomic ratio of $4.4\times10^{-6} \pm 2.2\times10^{-6}$. Selected values represent material at secular equilibrium, with the crustal $^{232}Th/^{238}U$ value of 3.8. *$\delta^{234}U = ([^{234}U/^{238}U]_{activity}-1) \times1000$. **$\delta^{234}U_{inital}$ are back-calculated to initial values based on respective U-Th corrected age. ***B.P. stands for "Before Present" where the "Present" is defined as the year 1950 CE.

DFT (cm)	^{238}U (ppb)	^{232}Th (ppt)	^{230}Th / ^{232}Th (atomic x10^{-6})	δ^{234}U* (measured)	^{230}Th / ^{238}U (activity)	^{230}Th Age (yr) (uncorrected)	$\delta^{234}U_{Initial}$** (corrected)	^{230}Th Age (yr) (corrected)	**^{230}Th Age (yr BP)*** (corrected BP)**
33.16	645±1.0	12060± 242	1606.6±32.5	671.1±1.9	1.8218±0.0052	349921±5500	1790.4±28.2	347724	**349654±5494**
35.68	488.0±0.7	332± 8	43637±985	638.3±1.9	1.7997±0.0049	369202±6292	1809.2±32.6	369193	**369123±6291**
37.48	483.9±0.6	82± 3	173203±7173	604.2±1.8	1.7725±0.0042	391522±6781	1824.1±35.4	391518	**391448±6782**
37.72	502.7±2.5	34± 1	430728±785	600.8±1.3	1.7578±0.0024	377947±3781	1745.5±19.0	377951	**377881±3782**
38.54	506.8±2.1	64± 1	227303±226	581.0±1.1	1.7471±0.0017	399250±3430	1792.8±17.7	399255	**399185±3430**
40.84	477.6±0.6	158± 4	84839±2265	551.0±1.7	1.7074±0.0037	399744±6917	1702.4±33.7	399738	**399668±6916**
42.36	648.3±0.9	2083± 42	8556±173	511.4±1.7	1.6673±0.0037	422222±8467	1683.5±40.6	422184	**422114±8465**
42.46	645.4±0.8	1643.6± 32.9	10784±216	515.0±1.9	1.6658±0.0025	410446±6331	1639.9±29.9	410415	**410345±6330**

Table S4: **Core P stable isotope results.** DFT= distance from top of core. $\delta^{18}O$ of core P were offset by up to 0.25 ‰ to align with the core D record (see methods).

MIS	DFT (cm)	$\delta^{18}O$ (‰)	$\delta^{13}C$ (‰)	Translation to Core D DFT (cm)	Adjusted $\delta^{18}O$ value (‰)
**MIS 5e**	3.66	-15.02	-2.16	7.85	-15.12
	3.68	-15.10	-2.20	7.93	-15.20
	3.70	-15.06	-2.22	8.01	-15.16
	3.72	-15.13	-2.30	8.09	-15.23
	3.74	-15.01	-2.23	8.17	-15.11
	3.76	-15.08	-2.26	8.25	-15.18
	3.78	-15.09	-2.28	8.33	-15.19
	3.80	-14.95	-2.32	8.41	-15.05
	3.82	-14.94	-2.31	8.49	-15.04
	3.84	-14.91	-2.34	8.57	-15.01
	3.86	-14.88	-2.33	8.65	-14.98
	3.88	-15.00	-2.36	8.74	-15.10
	3.90	-14.86	-2.34	8.82	-14.96
	3.92	-14.88	-2.40	8.90	-14.98
	3.94	-14.83	-2.43	8.98	-14.93
	3.96	-14.87	-2.45	9.06	-14.97
	3.98	-14.90	-2.46	9.14	-15.00
	4.00	-14.77	-2.45	9.22	-14.87
	4.02	-14.72	-2.45	9.30	-14.82
	4.04	-14.74	-2.50	9.38	-14.84
	4.06	-14.56	-2.52	9.46	-14.66
	4.08	-14.64	-2.55	9.54	-14.74
	4.10	-14.62	-2.60	9.62	-14.72
**MIS 7a**	10.48	-16.05	-1.99	17.40	-16.05
	10.50	-15.98	-1.98	17.68	-15.98
	10.52	-16.04	-1.99	17.97	-16.04
	10.54	-15.94	-1.98	18.25	-15.94
	10.56	-16.06	-2.02	18.53	-16.06
	10.58	-16.05	-1.99	18.81	-16.05
	10.60	-16.00	-2.01	19.09	-16.00
	10.62	-15.96	-2.00	19.38	-15.96
	10.64	-15.93	-1.99	19.66	-15.93
**MIS 7e**	11.30	-16.53	-2.12	20.97	-16.63
	11.32	-16.28	-2.15	21.01	-16.38
	11.34	-16.05	-2.12	21.06	-16.15
	11.36	-16.01	-2.17	21.10	-16.11
	11.38	-15.99	-2.08	21.15	-16.09
	11.40	-15.87	-2.07	21.19	-15.97
	11.42	-15.88	-2.12	21.24	-15.98
	11.44	-15.82	-2.14	21.28	-15.92
	11.46	-15.90	-2.12	21.33	-16.00
	11.48	-15.77	-2.12	21.37	-15.87
	11.50	-15.72	-2.11	21.42	-15.82
	11.52	-15.70	-2.16	21.47	-15.80
	11.54	-15.77	-2.13	21.51	-15.87
	11.56	-15.72	-2.10	21.56	-15.82
	11.58	-15.70	-2.11	21.60	-15.80
	11.60	-15.60	-2.13	21.65	-15.70
	11.62	-15.62	-2.14	21.69	-15.72
	11.64	-15.68	-2.13	21.74	-15.78
	11.66	-15.47	-1.95	21.78	-15.57
	11.68	-15.60	-2.07	21.83	-15.70
	11.70	-15.51	-1.96	21.87	-15.61
	11.72	-15.50	-1.98	21.92	-15.60
	11.74	-15.57	-2.04	21.97	-15.67
	11.76	-15.56	-2.07	22.01	-15.66
	11.78	-15.49	-2.04	22.06	-15.59
	11.80	-15.47	-2.06	22.10	-15.57
	11.82	-15.50	-2.07	22.15	-15.60
	11.84	-15.48	-2.07	22.19	-15.58

	11.86	-15.47	-2.07	22.24	-15.57
	11.88	-15.39	-2.05	22.28	-15.49
	11.90	-15.48	-2.15	22.33	-15.58
	11.92	-15.44	-2.14	22.37	-15.54
	11.94	-15.57	-2.20	22.42	-15.67
	11.96	-15.49	-2.17	22.47	-15.59
	11.98	-15.57	-2.26	22.51	-15.67
	12.00	-15.47	-2.23	22.56	-15.57
	12.02	-15.53	-2.27	22.60	-15.63
	12.04	-15.44	-2.25	22.65	-15.54
	12.06	-15.59	-2.28	22.69	-15.69
	12.08	-15.53	-2.28	22.74	-15.63
	12.10	-15.54	-2.31	22.78	-15.64
	12.12	-15.56	-2.32	22.83	-15.66
	12.14	-15.56	-2.35	22.87	-15.66
**MIS 9e**	19.24	-15.32	-1.92	30.74	-15.57
	19.26	-15.31	-1.91	31.01	-15.56
	19.28	-15.30	-1.92	31.27	-15.55
	19.30	-15.38	-1.94	31.53	-15.63
	19.32	-15.32	-1.90	31.79	-15.57
	19.34	-15.32	-1.98	32.06	-15.57

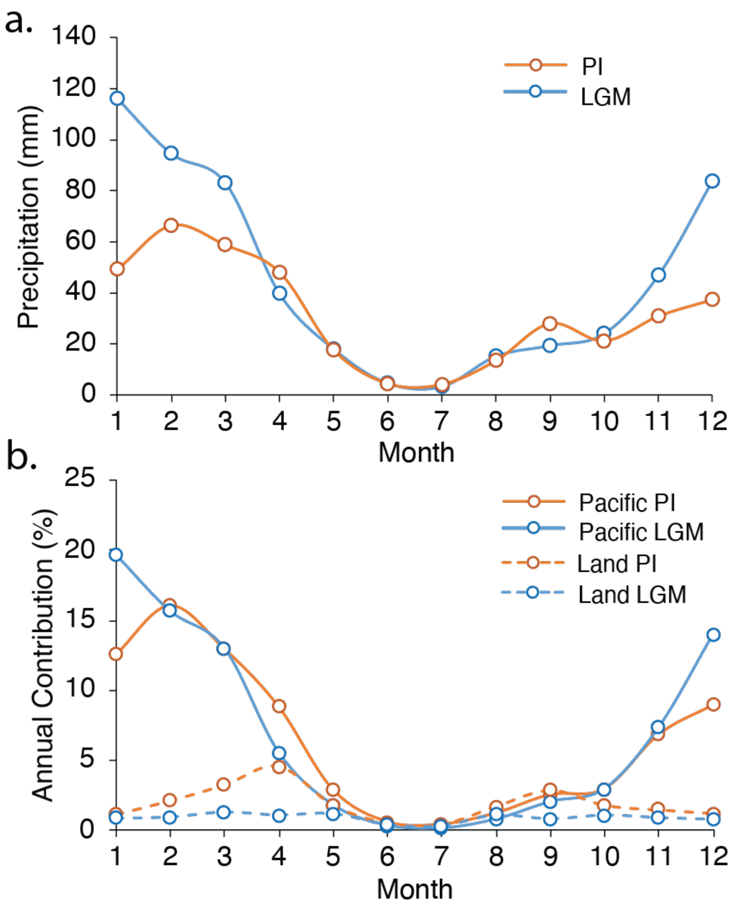

Figure S4: iCESM last glacial maximum (LGM) and preindustrial (PI) monthly change in precipitation. A: LGM versus PI precipitation amount. B: Annual percent contribution of monthly rainfall sourced from the Pacific and North American continent (abbreviated as Land).

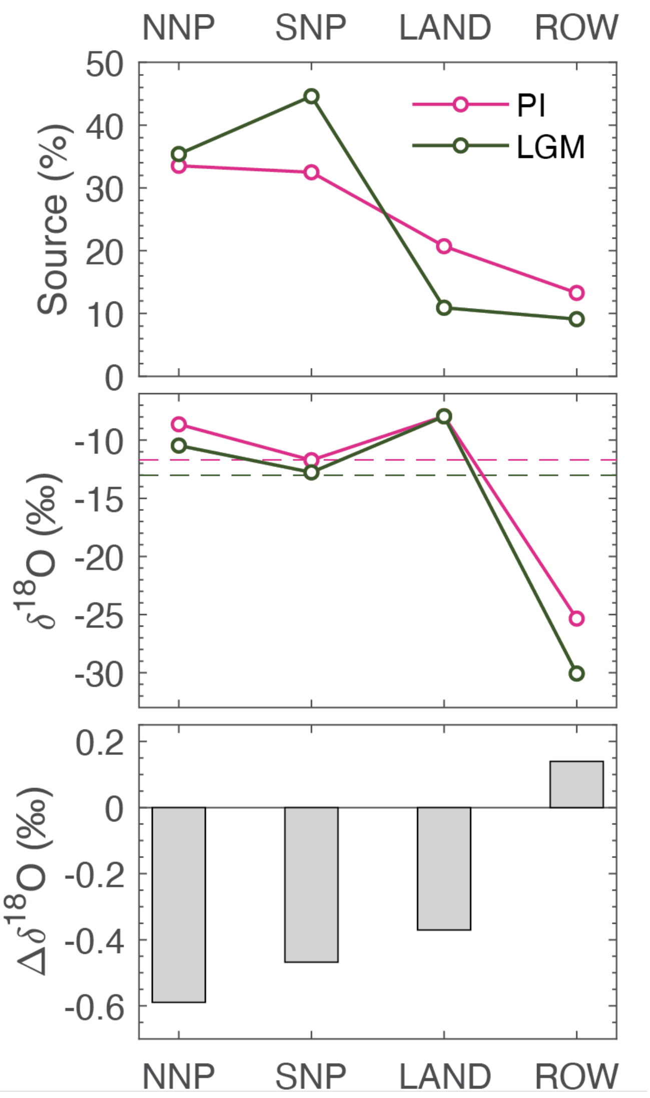

Figure S5: iCESM water tagging results showing the LGM-PI change in percent contribution, $\delta^{18}O$, and $\Delta\ \delta^{18}O$ of moisture sourced from the northern North Pacific (NNP), southern North Pacific (SNP), the North American continent (LAND), and rest of the world (ROW). Source divisions shown in (*7*). Dashed lines represent average.

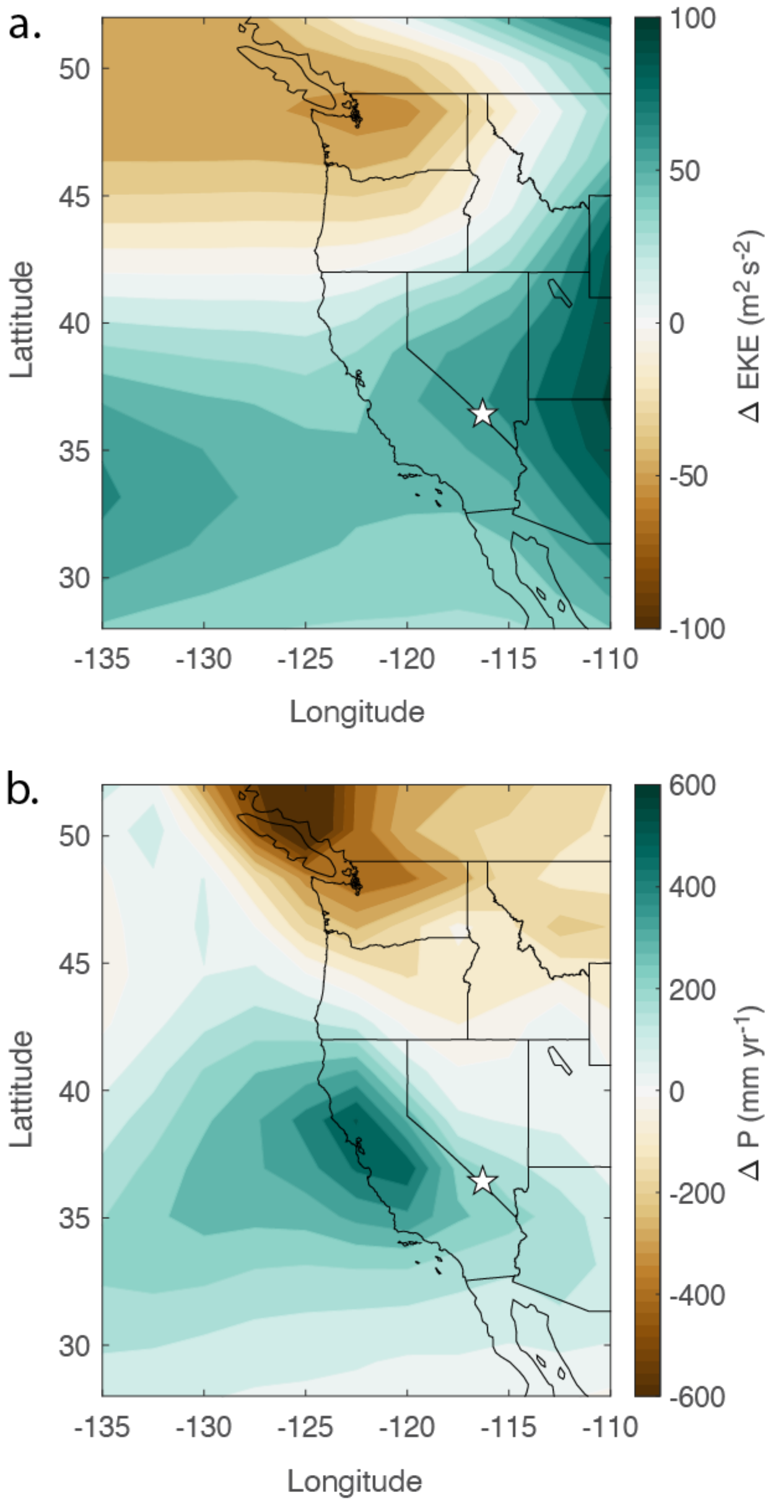

Figure S6: A: iCESM LGM-PI difference in mean eddy kinetic energy (EKE) at the 600 mbar atmospheric level. B: iCESM LGM-PI difference in mean precipitation (P). White star shows location of Devils Hole caves.

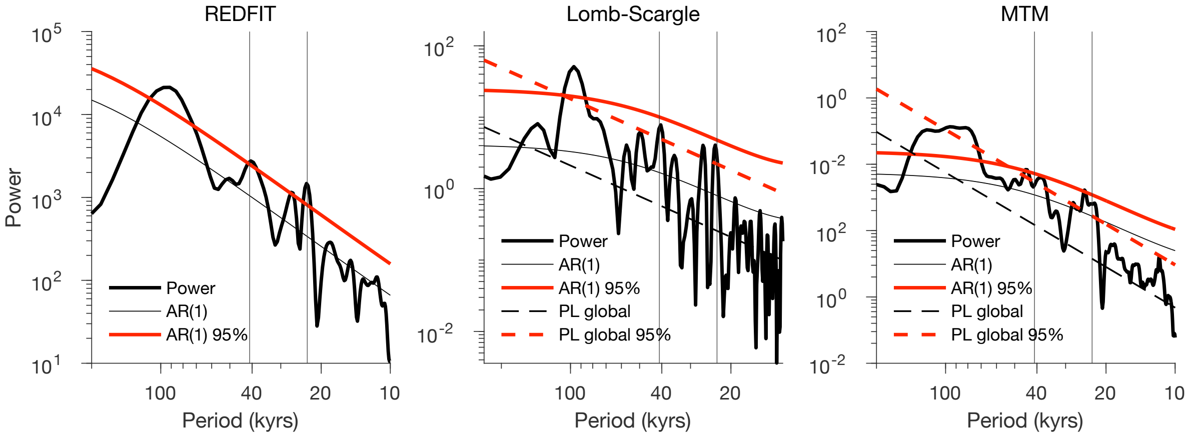

Figure S7: DH2 $\delta^{18}O$ autospectrum. Power spectrum for DH2 $\delta^{18}O$ computed using the REDFIT method (*8, 9*), the Lomb-Scargle method (*10, 11*) and the Multi-Taper method (MTM) (*12*). REDFIT (*8*) and Acycle (*13*) software were used for spectral analysis, red noise and 95% significance threshold computation. AR(1) stands for first order autoregression (*8, 9, 14, 15*). PL stands for Power Law. In some cases spectral analysis on simulated data generated from a noise process with a power law (f^{-1}) spectrum matches better to the data than the autoregression model (*16*).

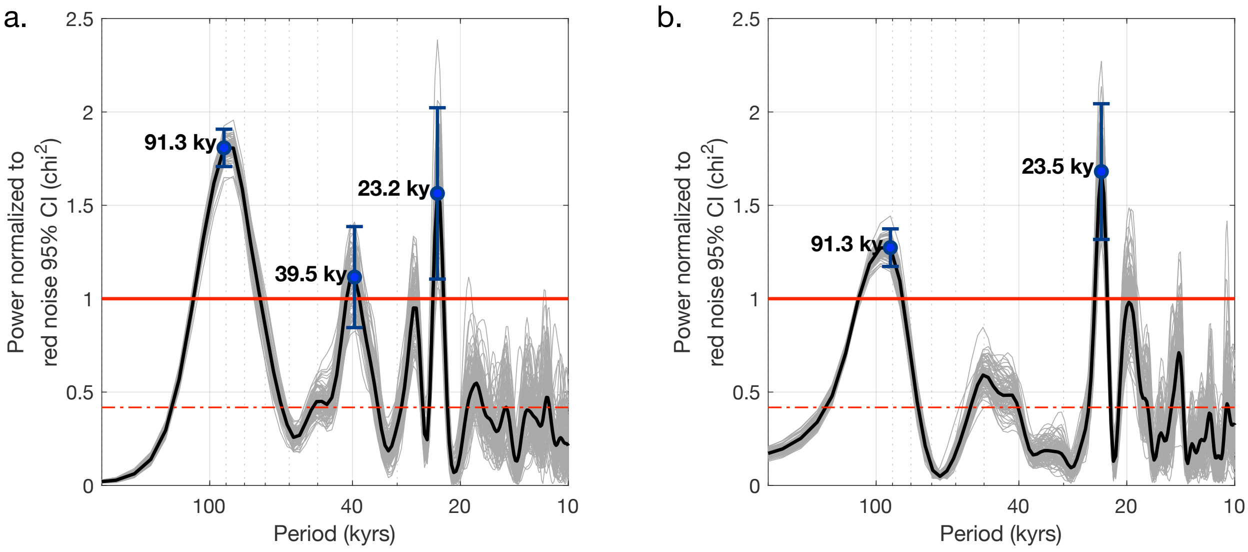

Figure S8. DH2 power spectrum of 100 OxCal age model $\delta^{18}O$ (a) and $\delta^{13}C$ (b) records, computed using REDFIT freeware (Lomb-Scargle method with Welch-Overlapped-Segment-Averaging; number of overlapping windows = 2) (see methods). Prior to spectral analysis, each record has been binned and interpolated to 1000-yr resolution to remove higher frequency signals. Each power spectrum has been normalized to its corresponding 95% false alarm record, such that power >1 is interpreted as a frequency significant above red noise. The black line is the mean normalized power of the 100 records calculated at each frequency. The blue circles highlight local maximum mean normalized power above 1, with 2σ error bars. The red dash dot line represents the theoretical red noise (first-order autoregressive process, AR(1)).

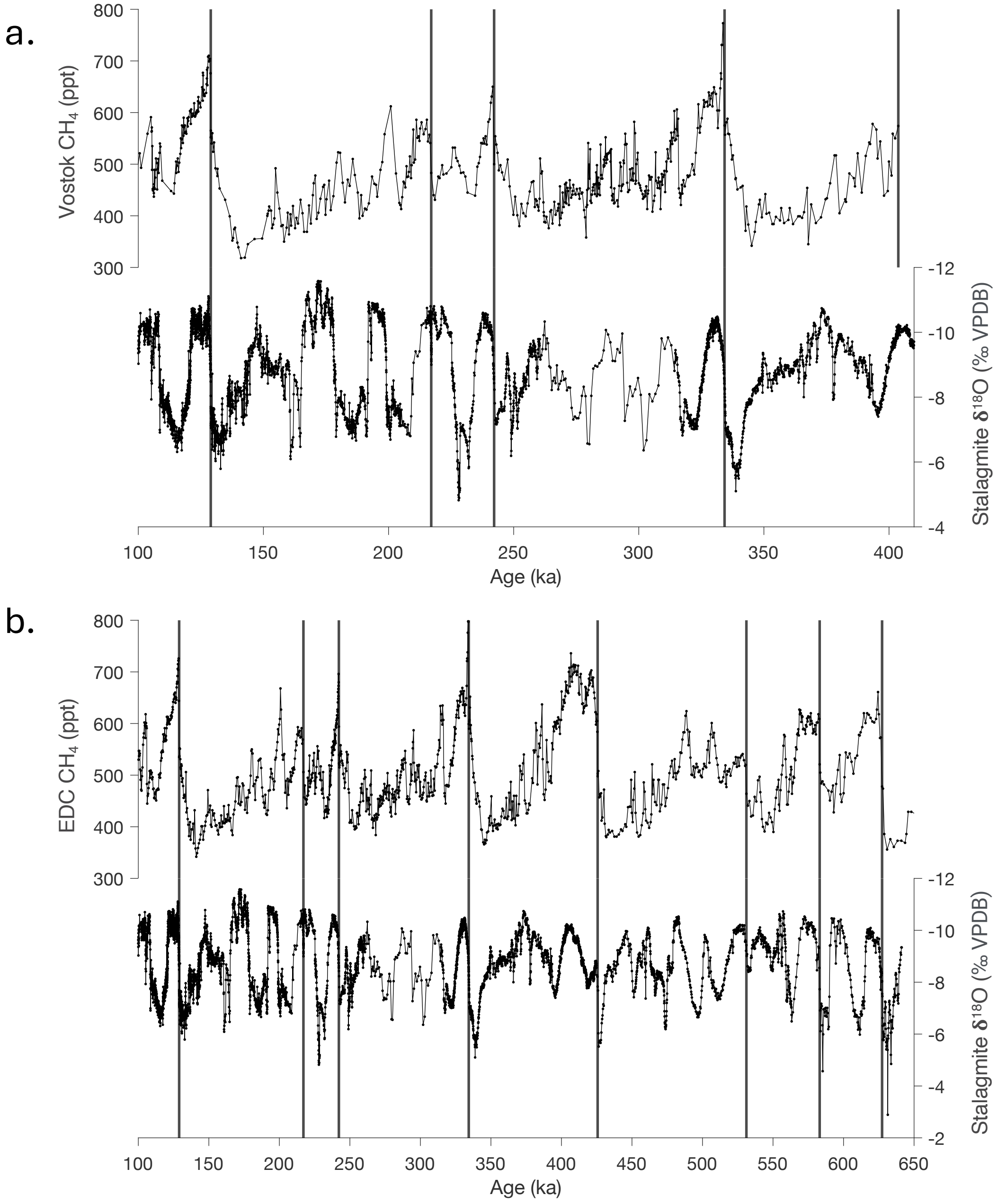

Figure S9: A: comparison between tuned Vostok CH_4 record (*17*) on the AICC2012 age model and the Chinese stalagmite $\delta^{18}O$ record (*18*). B: comparison between tuned Dome C (EDC) CH_4 record (*19*) and the Chinese stalagmite $\delta^{18}O$ record (*18*). Tie points shown with vertical black lines. Tuning done by QAnalyseries computing (*20, 21*).

Table S5: Tie points between the Vostok CH_4 record (*17*) on the AICC2012 age model and the Asian Monsoon (AM) stalagmite $\delta^{18}O$ record (*18*), and the Dome C (EDC) CH_4 record (*19*) on the AICC2012 age model (*22*) and the AM stalagmite $\delta^{18}O$ record (*18*), and between, for the interval 600-207 ka.

Atmospheric CH_4 – Asian Monsoon Tie Points

Vostok

Tie points	CH4 depth	CH4 age*	AM age	Event
0	173.1	3662		top
15	1883.3	129117	128957	TII
16	2649.4	218066	217060	TIIIa
17	2780	243688	242191	TIII
18	3126.5	335705	334281	TIV
19	3270.6	403716		end

*AICC2012 age

EDC

Tie points	CH4 depth	CH4 age*	AM age	Event
0	99.34	194		top
15	1724.27	128880	128957	TII
16	2194.52	217505	217060	TIIIa
17	2307.23	243337	242191	TIII
18	2523.4	335388	334281	TIV
19	2781.21	426172	425668	TV
20	2907.32	532559	531141	TVI
21	2997.99	580385	583089	TVIIa
22	3038.22	625338	627336	TVII
23	3190.53	799472		end

*AICC2012 age

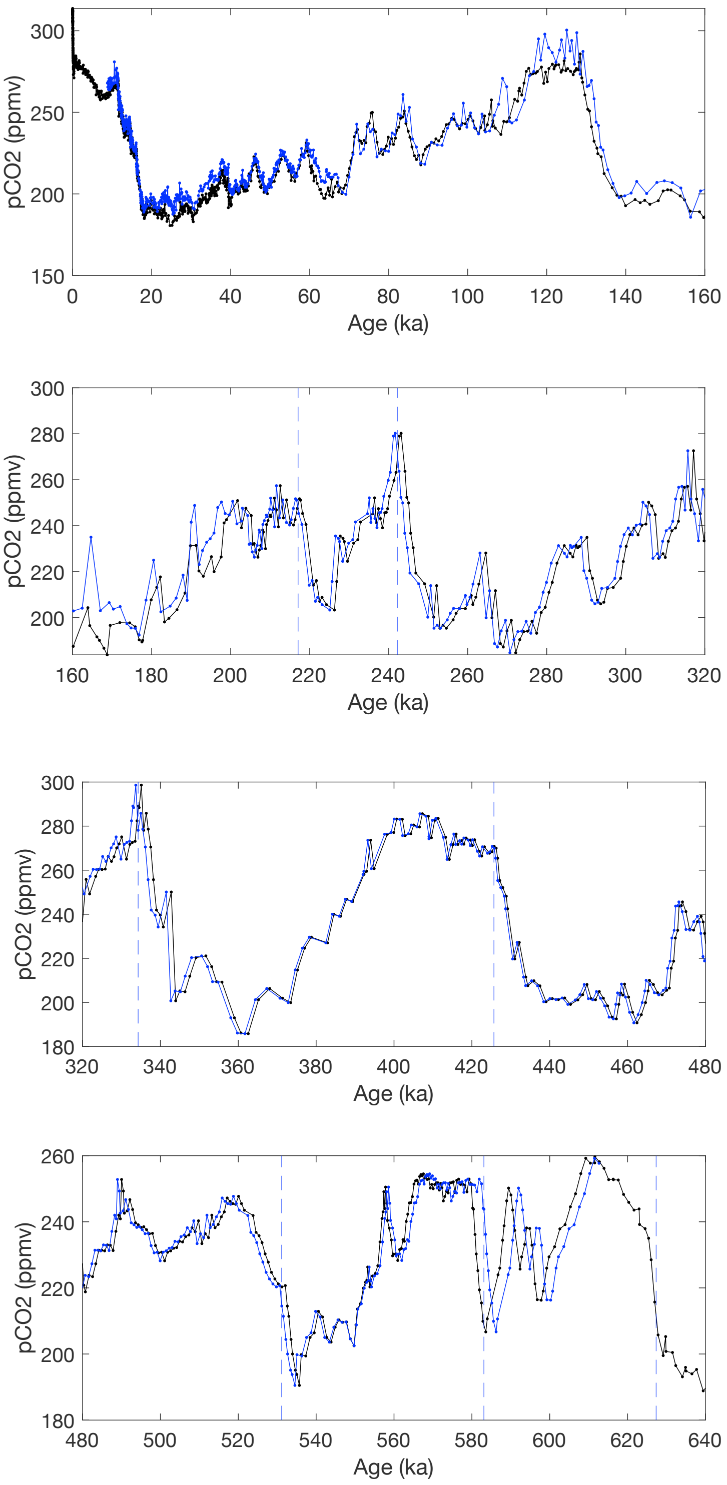

Figure S10: Composite CO_2 record age model comparison. Blue line: this study's 600-207 ka AICSTAL2024 chronology (see text) combined with WD2014 (60-0 ka; *23*) and DF2021 (67-207 ka; *24*). Black: Bereiter,

Eggleston, Schmitt, Nehrbass-Ahles, Stocker, Fischer, Kipfstuhl and Chappellaz (*25*) composite CO_2 age model on the AICC2012 chronology (*22*).

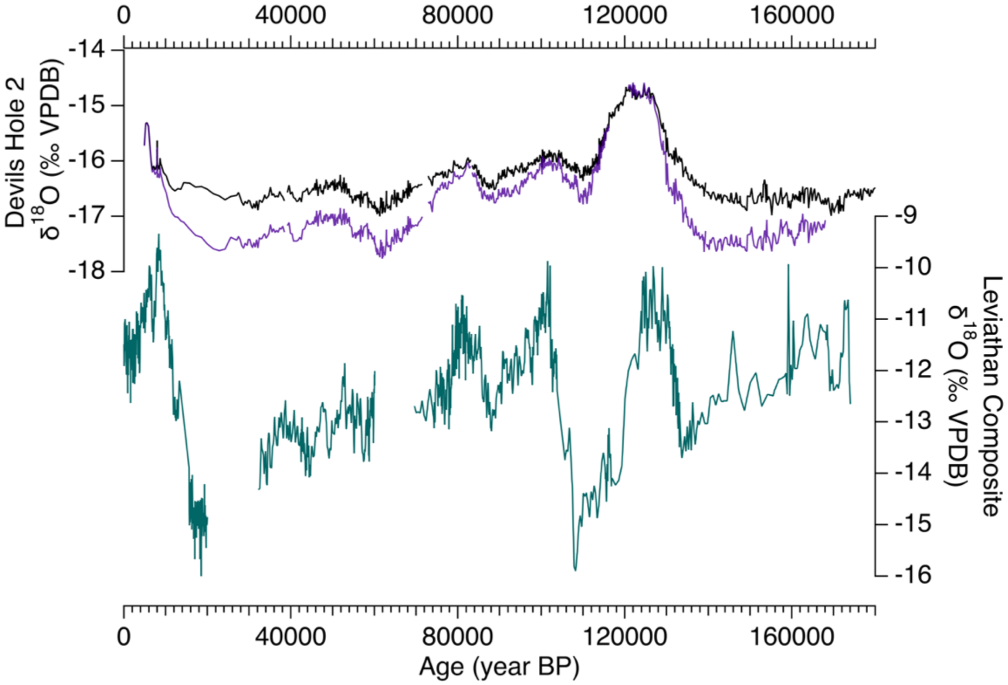

Figure S11: Great Basin speleothem $\delta^{18}O$ records. Devils Hole 2 $\delta^{18}O$ in black (*2*) and with ice volume $\delta^{18}O$ correction in purple (*2*), and the ice-volume corrected $\delta^{18}O$ record from Nevada stalagmites in teal (*26, 27*).

Text S1: As shown in Fig. S11 and discussed in previous publications, the amplitude of Devils Hole 2 $\delta^{18}O$ is less than half of that of the Leviathan $\delta^{18}O$. This has been previously attributed to the slow rate of carbonate deposition (*2*) and aquifer averaging (*27*) resulting in a more muted response of $\delta^{18}O$ that is representative of a larger spatial area relative to stalagmites which grow from infiltrating surface waters Cave temperatures can also effect the amplitude of $\delta^{18}O$ through its control on equilibrium fractionation between water and calcite (*28*). Triple oxygen isotopes have also shown sensitivity to mineralization temperature at Leviathan and evaporation at Lehman caves (*29*). Lastly, back trajectory analysis of modern seasonal rainfall by Cross (2015) (see figure 24) show that Leviathan and Lehman Caves receive a greater proportion of precipitation sourced from continental land, which according to iCESM simulations from this study are enriched relative to Pacific moisture.

Table S6: Phasing of multiple climate records (≥500 ka) relative to DH2 $\delta^{18}O$. Maximum correlation coefficients (r) using a lagged correlation method (see methods), with negative values indicating that DH2 $\delta^{18}O$ leads. "CO_2 #1" is the atmospheric CO_2 composite (*17, 30-33*) on the ice core AICC2023 chronology (*34*). "CO_2 #2" is the CO_2 composite on the AICSTAL chronology (this study). "Pac SST" is a tropical east Pacific SST record (*35*) tuned to LR04 (*36*). "Benthic" is the global benthic $\delta^{18}O$ curve on the LR04 chronology (*36*). "RSL #1" is an absolute-dated sea level curve (*37*). "RSL #2" a global RSL stack (*38*) tuned to the LR04 chronology (*36*). Correlation coefficients are calculated in MatLab, p-values <0.01×10^{-10}

	**CO_2 #1**	**CO_2 #2**	**Pac SST**	**Benthic**	**RSL #1**	**RSL#2**
**Max. correlation (r) to DH2 $\delta^{18}O$**	0.87242	0.87215	0.70341	0.8177	0.76903	0.71932
**Phasing relative to DH2 $\delta^{18}O$ (years)**	200	-300	-700	-2250	-1650	-5400

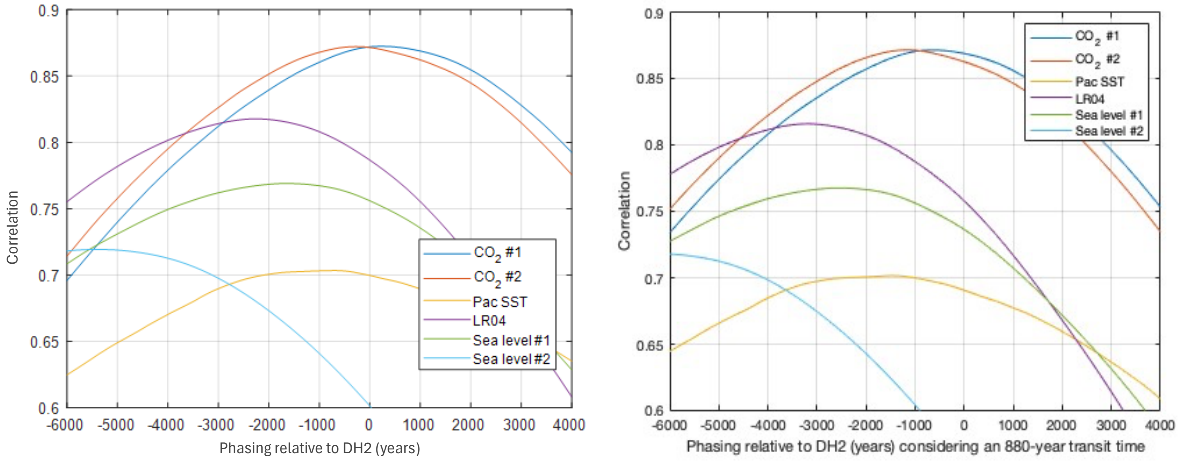

Figure S12: Probability density curves fitted by the Gaussian distribution of several climate records (see Table S5) and their respective phasing relative to DH2 $\delta^{18}O$. Negative values indicate that DH2 $\delta^{18}O$ leads, positive values indicate that DH2 $\delta^{18}O$ lags. Right panel: as in left, but including an 880-year groundwater transit time (i.e. adding 880 years) to DH2 $\delta^{18}O$.

Table S7: Midpoint analysis results for Devils Hole $\delta^{18}O$ and select climate records, as in table S6, "CO_2 #1" is the atmospheric CO_2 composite (*17, 30-33*) on the ice core AICC2023 chronology (*34*). "CO_2 #2" is the CO_2 composite on the AICSTAL chronology (this study). "Pac SST" is a tropical east Pacific SST record (*35*) tuned to LR04 (*36*). "Benthic" is the global benthic $\delta^{18}O$ curve on the LR04 chronology (*36*). "RSL #1" is an absolute-dated sea level curve (*37*). "RSL #2" a global RSL stack (*38*) tuned to the LR04 chronology (*36*).

Record	Termination	Midpoint Age (yrs BP)	Midpoint proxy value	Offset from DH2 midpoint (yrs)*
Devils Hole	TII	132152	-15.80 ‰	
	TIII	244027	-16.16 ‰	
	TIV	341120	-16.23 ‰	
	TV	429843	-16.28 ‰	
CO2 #1 AICC2023	TII	132188	236.7 ppm	-36
	TIII	244990	237.8 ppm	-963
	TIV	341210	249.7 ppm	-90
	TV	429484	234.5 ppm	359
CO2 #2 AICSTAL	TII	132688	236.7 ppm	-536
	TIII	244026	237.8 ppm	1
	TIV	341515	249.7 ppm	-395
	TV	429299	234.5 ppm	543
Sea Level #1	TII	132625	-46.1 m	-473
	TIII	241625	-49.4 m	2402
	TIV	333875	-59.2 m	7245
	TV	429250	-67.4 m	593
Sea Level #2	TII	130610	-62.1 m	1542
	TIII	246840	-51.7 m	-2813
	TIV	335198	-52.3 m	5922
	TV	419350	-52.7 m	10492
Benthic d18O	TII	129333	4.1 ‰	2819
	TIII	241741	4.0 ‰	2286
	TIV	333303	4.0 ‰	7817
	TV	421974	4.1 ‰	7869
Tropical East Pacific SSTs	TII	130831	25.6 °C	1321
	TIII	241716	24.9 °C	2311
	TIV	345100	25.3 °C	-3980
	TV	421765	25.0 °C	8077

***DH2 midpoint - climate record midpoint; negative values indicate DH2 $\delta^{18}O$ lags**

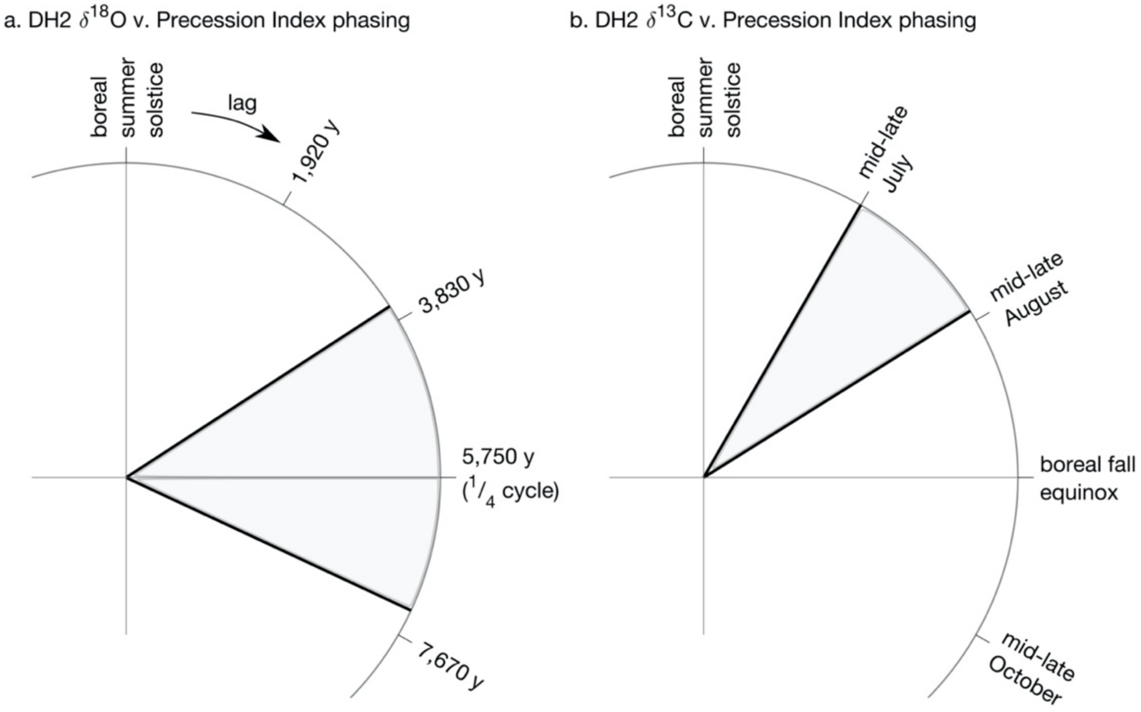

Figure S13: Phase lag relative to the orbital precession index for DH2 $\delta^{18}O$ (a) and $\delta^{13}C$ (b) (DH2 record on the mean age model), shown on phase wheels. Zero phase (pointing up) is set as alignment with precession index minimum, equivalent to the Northern Hemisphere summer solstice (June 21st) insolation maximum. Note, the Gregorian calendar date of June 21st for boreal summer solstice is approximate due to variable season length set by the precession index. In (a), phase wheel tick marks note years of lag from precessional index minimum (in a 23,000 year cycle, a $1/4^{th}$ cycle lag is equivalent to 5,750 years lag). Arrows mark the direction of increasing years of lag from the set zero phase. In (b), phase wheel tick marks note alignment with other points within the precessional cycle: pointing up is alignment with boreal summer solstice, while pointing to the right is alignment with boreal fall equinox. A record's alignment with boreal fall equinox is equivalent to 5,750 years lag behind boreal summer solstice in the precessional index.

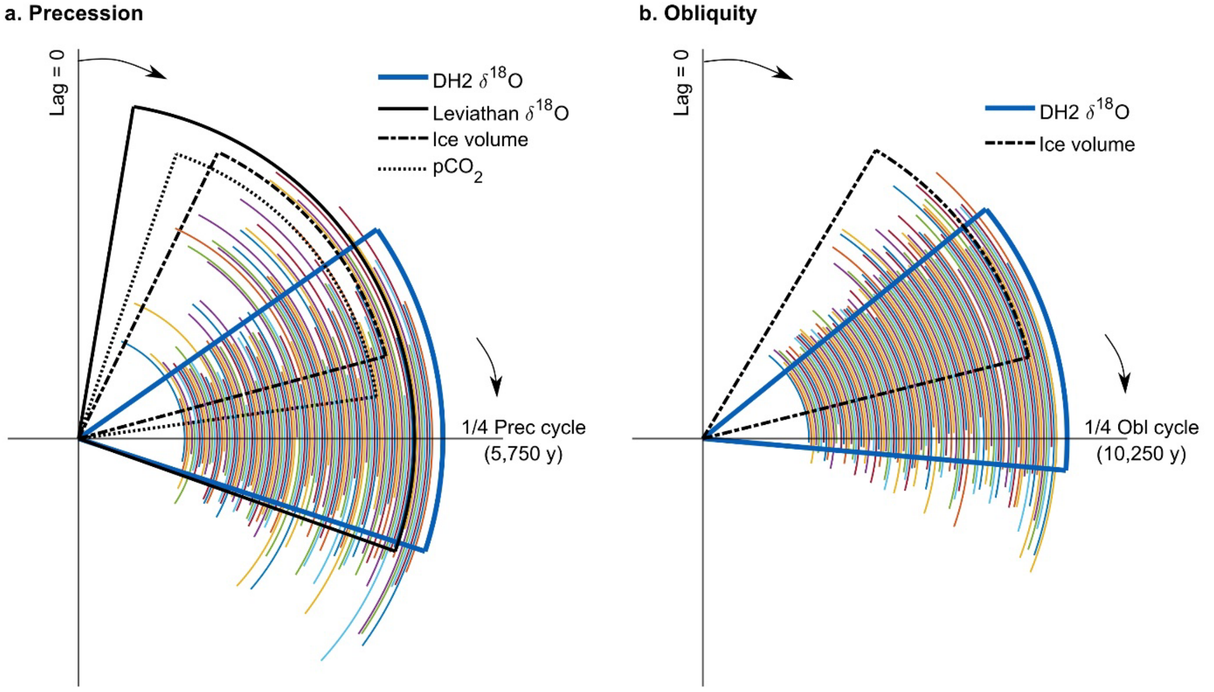

Figure S14: Devils Hole 2 (DH2) δ^{18}O, Leviathan δ^{18}O, ice volume, and pCO_2 phase lag relative to the orbital precession index (a) and obliquity (b). In (a), zero phase (pointing up) is set as precession index minimum, equivalent to the Northern Hemisphere summer solstice (June 21st) insolation maximum, and arrows mark direction of increasing years of lag from the set zero phase (1 cycle = 23 kyr). In (b), zero phase (pointing up) is set as maximum tilt, and arrows mark direction of increasing years of lag from the set zero phase (1 cycle = 41 kyr). In (a), phase lag 95% CI (wedge arcs) are relative to the orbital precession index, in the 22-24 kyr period window. In (b), phase lag 95% CI (wedge arcs) are relative to orbital obliquity, in the 38-43 kyr period window. Wedges are staggered in height for easier viewing. Dataset sources: Stacked colored lines demonstrate Devils Hole 2 (DH2) δ^{18}O phase variability due to age model uncertainty (phase lags calculated from 100 individual records are shown). A bold blue line wedge shows the phase lag calculated using the DH2 δ^{18}O record on its mean age model. The phase lag for the Leviathan δ^{18}O record with the precessional index minimum (600 to 7000 years) is plotted as published in Lachniet, Asmerom, Polyak and Denniston (*27*). Global ice volume is inferred from the absolute-dated Red Sea RSL record (*37*)). The atmospheric CO_2 record (*25*) is on the AICC2023 chronology (*34*). All records used in cross correlation analysis have age models independent of orbital tuning.

References

1. K. J. Halford, T. R. Jackson, "Groundwater characterization and effects of pumping in the Death Valley regional groundwater flow system, Nevada and California, with special reference to Devils Hole," (US Geological Survey, 2020).
2. G. E. Moseley *et al.*, Reconciliation of the Devils Hole climate record with orbital forcing. *Science* **351**, 165-168 (2016).
3. K. A. Wendt *et al.*, Paleohydrology of southwest Nevada (USA) based on groundwater 234U/238U over the past 475 ky. *GSA Bulletin* **132**, 793-802 (2020).
4. X. Li *et al.*, Novel method for determining 234 U-238 U ages of Devils Hole 2 cave calcite. *Geochronology Discussions* **2020**, 1-16 (2020).
5. H. Cheng *et al.*, Improvements in 230Th dating, 230Th and 234U half-life values, and U–Th isotopic measurements by multi-collector inductively coupled plasma mass spectrometry. *Earth and Planetary Science Letters* **371**, 82-91 (2013).
6. A. Jaffey, K. Flynn, L. Glendenin, W. t. Bentley, A. Essling, Precision measurement of half-lives and specific activities of U 235 and U 238. *Physical review C* **4**, 1889 (1971).
7. C. He *et al.*, Abrupt Heinrich Stadial 1 cooling missing in Greenland oxygen isotopes. *Science advances* **7**, eabh1007 (2021).
8. M. Schulz, M. Mudelsee, REDFIT: estimating red-noise spectra directly from unevenly spaced paleoclimatic time series. *Computers & Geosciences* **28**, 421-426 (2002).
9. M. Schulz, K. Stattegger, SPECTRUM: Spectral analysis of unevenly spaced paleoclimatic time series. *Computers & Geosciences* **23**, 929-945 (1997).
10. N. R. Lomb, Least-squares frequency analysis of unequally spaced data. *Astrophysics and space science* **39**, 447-462 (1976).
11. J. D. Scargle, Studies in astronomical time series analysis. II-Statistical aspects of spectral analysis of unevenly spaced data. *Astrophysical Journal, Part 1, vol. 263, Dec. 15, 1982, p. 835-853.* **263**, 835-853 (1982).
12. D. J. Thomson, Spectrum estimation and harmonic analysis. *Proceedings of the IEEE* **70**, 1055-1096 (1982).
13. M. Li, L. Hinnov, L. Kump, Acycle: Time-series analysis software for paleoclimate research and education. *Computers & Geosciences* **127**, 12-22 (2019).
14. M. Mudelsee, Ramp function regression: a tool for quantifying climate transitions. *Computers & Geosciences* **26**, 293-307 (2000).
15. M. E. Mann, J. M. Lees, Robust estimation of background noise and signal detection in climatic time series. *Climatic change* **33**, 409-445 (1996).
16. S. Vaughan, R. Bailey, D. Smith, Detecting cycles in stratigraphic data: Spectral analysis in the presence of red noise. *Paleoceanography* **26**, (2011).
17. J.-R. Petit *et al.*, Climate and atmospheric history of the past 420,000 years from the Vostok ice core, Antarctica. *Nature* **399**, 429-436 (1999).
18. H. Cheng *et al.*, The Asian monsoon over the past 640,000 years and ice age terminations. *Nature* **534**, 640-646 (2016).
19. L. Loulergue *et al.*, Orbital and millennial-scale features of atmospheric CH4 over the past 800,000 years. *Nature* **453**, 383-386 (2008).
20. S. Kotov, H. Pälike, in *AGU Fall Meeting Abstracts*. (2018), vol. 2018, pp. PP53D-1230.
21. D. Paillard, L. Labeyrie, P. Yiou, Macintosh program performs time-series analysis. *Eos, Transactions American Geophysical Union* **77**, 379-379 (1996).

22. L. Bazin *et al.*, An optimized multi-proxy, multi-site Antarctic ice and gas orbital chronology (AICC2012): 120–800 ka. *Climate of the Past* **9**, 1715-1731 (2013).
23. C. Buizert *et al.*, The WAIS Divide deep ice core WD2014 chronology–Part 1: Methane synchronization (68–31 ka BP) and the gas age–ice age difference. *Climate of the Past* **11**, 153-173 (2015).
24. I. Oyabu *et al.*, The Dome Fuji ice core DF2021 chronology (0–207 kyr BP). *Quaternary Science Reviews* **294**, 107754 (2022).
25. B. Bereiter *et al.*, Revision of the EPICA Dome C CO2 record from 800 to 600 kyr before present. *Geophysical Research Letters* **42**, 542-549 (2015).
26. M. S. Lachniet, R. F. Denniston, Y. Asmerom, V. J. Polyak, Orbital control of western North America atmospheric circulation and climate over two glacial cycles. *Nature communications* **5**, 3805 (2014).
27. M. Lachniet, Y. Asmerom, V. Polyak, R. Denniston, Arctic cryosphere and Milankovitch forcing of Great Basin paleoclimate. *Scientific Reports* **7**, 12955 (2017).
28. S.-T. Kim, J. R. O'Neil, Equilibrium and nonequilibrium oxygen isotope effects in synthetic carbonates. *Geochimica et cosmochimica acta* **61**, 3461-3475 (1997).
29. T. E. Huth *et al.*, A framework for triple oxygen isotopes in speleothem paleoclimatology. *Geochimica et Cosmochimica Acta* **319**, 191-219 (2022).
30. T. K. Bauska, S. A. Marcott, E. J. Brook, Abrupt changes in the global carbon cycle during the last glacial period. *Nature Geoscience* **14**, 91-96 (2021).
31. B. Bereiter *et al.*, Mode change of millennial CO2 variability during the last glacial cycle associated with a bipolar marine carbon seesaw. *Proceedings of the National Academy of Sciences* **109**, 9755-9760 (2012).
32. R. Schneider, J. Schmitt, P. Köhler, F. Joos, H. Fischer, A reconstruction of atmospheric carbon dioxide and its stable carbon isotopic composition from the penultimate glacial maximum to the last glacial inception. *Climate of the Past* **9**, 2507-2523 (2013).
33. U. Siegenthaler *et al.*, Stable carbon cycle climate relationship during the Late Pleistocene. *Science* **310**, 1313-1317 (2005).
34. M. Bouchet *et al.*, The Antarctic Ice Core Chronology 2023 (AICC2023) chronological framework and associated timescale for the European Project for Ice Coring in Antarctica (EPICA) Dome C ice core. *Climate of the Past* **19**, 2257-2286 (2023).
35. K. A. Dyez, R. Zahn, I. R. Hall, Multicentennial Agulhas leakage variability and links to North Atlantic climate during the past 80,000 years. *Paleoceanography* **29**, 1238-1248 (2014).
36. L. E. Lisiecki, M. E. Raymo, A Pliocene-Pleistocene stack of 57 globally distributed benthic δ18O records. *Paleoceanography* **20**, (2005).
37. K. M. Grant *et al.*, Sea-level variability over five glacial cycles. *Nature Communications* **5**, 5076 (2014).
38. R. M. Spratt, L. E. Lisiecki, A Late Pleistocene sea level stack. *Climate of the Past* **12**, 1079-1092 (2016).
