# Supplementary material for: Controls on the southwest USA hydroclimate over the last six glacial-interglacial cycles
Source: Nat Commun. 2025 Nov 14;16:10007. doi: 10.1038/s41467-025-64963-1 (PMC12618935; doi:10.1038/s41467-025-64963-1)
Supplement: Supplementary file 2 — Description of Additional Supplementary Files [file 41467_2025_64963_MOESM2_ESM.pdf]

Description of Additional Supplementary Files

Supplementary Data 1: Signal-to-noise ratios of SomaScan assay

Supplementary Data 2: Coefficients and p-value of age and sex in linear model predicting somamer level in healthy control (HC) cohort used for HC age-/sex-adjustment

Supplementary Data 3: Master dataset – associations of somamers/pathways with clinical/demographic outcomes

Supplementary Data 4: Enrichment of cell-specific markers associated with various aspects of multiple sclerosis

Supplementary Data 5: Ingenuity Pathway Analysis results for Canonical Pathways

Supplementary Data 6: Ingenuity Pathway Analysis results for Upstream regulators

Supplementary Data 7: Ingenuity Pathway Analysis results for Causal networks

Supplementary Data 8: Ingenuity Pathway Analysis results for Diseases and Functions

Supplementary Data 9: Composition of Ingenuity Pathway Analysis Pathway/MS vs HC activation scores

Supplementary Data 10: Composition of Ingenuity Pathway Analysis Pathways/Multiple Sclerosis vs Healthy controls activation scores with protein directionality

Supplementary Data 11: Predictors of the Elastic Net model of the number of contrast-enhancing lesion from cerebrospinal fluid biomarkers

Supplementary Data 12: Results from STRING analysis of the 162 cerebrospinal fluid predictors of the number of contrast-enhancing lesion in the Elastic Net model
